# Bibliometric and visual analysis of palliative care in colorectal cancer research from 2015 to April 2025

**DOI:** 10.1186/s41065-025-00559-5

**Published:** 2025-09-26

**Authors:** Xiaohui Zhang, Liangliang Li, Anxia Li

**Affiliations:** 1 Department of Pharmacy, The Second Affiliated Hospital of Guangxi Medical University, Nanning, 530007 Guangxi China; 2 Department of Pharmacy, Sanya Central Hospital (The Third People’s Hospital of Hainan Province), Sanya, 572000 Hainan China; 3 Guangxi International Zhuang Medical Hospital, Nanning, 530001 Guangxi China

**Keywords:** Bibliometrics, Colorectal cancer, Palliative care, Cancer, Tumor therapy

## Abstract

**Objective:**

Palliative care, as a treatment method for incurable diseases, can alleviate patients’ pain and improve their quality of life(QOL) to a certain extent. This treatment method is also common in the treatment of advanced malignant tumors. This study aims to examine the current research status and hotspots of palliative care in colorectal cancer(CRC) from January 1, 2015, to April 15, 2025, providing reference value for future research.

**Methods:**

Based on the Web of Science Core Collection (WoSCC) database, we retrieved articles related to palliative care in CRC published from January 1, 2015, to April 15, 2025, and analyzed them by using R software, VOS viewer and CiteSpace.

**Results:**

This study included a total of 1146 relevant articles, which were published in 363 academic journals. Between 2015 and 2016, the number of studies focusing on palliative care in CRC has shown an upward trend. From 2016 to 2024, publication volume was relatively stable, with a slight peak observed in 2020. The top five countries in terms of publication volume were The United States, China, Netherlands, The United Kingdom, Australia. Among them, Australia was the leading country based on the multi-cooperation. The most frequently published journal was *Supportive Care in Cance*r, while *The Journal of Clinical Oncology* held a significant advantage in citation frequency. Our study demonstrates that research primarily focusing on “Precision and individualized systemic palliative treatment strategies”, “Palliative management of local complications and surgical interventions” and “Multidimensional supportive care” are getting increasing attention, indicating that the research tends to be diversified in the future.

**Conclusion:**

Palliative care not only improves quality of life but also represents a growing area of research interest in CRC. This study provides a systematic review and multi-dimensional analysis of the current research status and hotspots in palliative care cancer, aiming to offer valuable insights for future research.

**Supplementary Information:**

The online version contains supplementary material available at 10.1186/s41065-025-00559-5.

## Introduction

Colorectal cancer(CRC) is one of the most common gastrointestinal tumors, posing a serious threat to human health. According to the Global Cancer Statistics report of 2024 [1], there were nearly 20 million new patients of cancer worldwide in 2022, of which CRC accounted for 9.6%. Approximately 9.7 million people died from the disease, with a mortality of 9.3%. The morbidity of CRC ranked third, and its mortality ranked second. In the report, the mortality of CRC was rising, followed by lung cancer (18.7%). In terms of gender, the mortality and morbidity of CRC among male patients were also at the forefront. Clearly, CRC has developed into a major public health concern, endangering human health. Currently, the main treatment methods for CRC include surgery, radiotherapy, chemotherapy, targeted drug therapy, immunotherapy, and palliative chemotherapy [2, 3]. The survival rate of CRC was closely related to its clinical stage. A study revealed that 5-year survival rate of stage I patients could reach more than 90%, while that of stage IV patients dropped to 14% [4]. Another study showed that surgical therapy was the primary treatment for patients with stageII and III of CRC, with 5-year overall survival rates as high as 80% and 60%, respectively [5]. However, in reality, not all patients have the opportunity for surgery. Moreover, the use of treatments such as radiotherapy, systemic chemotherapy, and targeted therapies may cause such patients to experience persistent pain, physiological limitations and negative emotions, which could significantly reduce their health-related quality of life(QOL) [6]. Therefore, it is urgently necessary to improve QOL for patients undergoing cancer.

Palliative care is an approach that focuses on relieving patients’ suffering and improving their QOL. It provides treatment on physiological, emotional, social, and spiritual levels for patients with incurable or uncontrollable disease progression through early identification and accurate assessment, helping patients achieve a better physical and mental state, thereby improving their QOL [7]. Palliative care is patient-oriented and suitable for individuals of any age and at any stage of cancer. It does not interfere with curative treatments, and can be used simultaneously [8]. A study found that palliative care was beneficial in changing the cost of cancer treatment [9]. Patients with advanced CRC often experience intestinal obstruction [10, 11], bleeding [12], and pain. Palliative surgery, radiotherapy, and chemotherapy can help relieve obstruction [13] and pain, thereby reducing the patient’s discomfort. Additionally, palliative care can assist patients in living comfortably within their limited lifespan by correcting malnutrition, providing psychological interventions, and relieving anxiety [14]. One of studies has mentioned that the QOL years in palliative patients with CRC is similar to that of people who are no longer suffering from the disease [15, 16]. For advanced, incurable CRC, palliative care may be able to prolong survival time to some extent. However, the wishes of the patient and family need to be respected, with a focus on QOL [17]. Despite considerable research in this field, there is a lack of studies that systematically review the benefits, hotspots, and future directions of palliative care in CRC research from January 1, 2015, to April 15, 2025 through bibliometrics and visual analysis. To a certain extent, it affects the development of this research.

Bibliometrics, a tool based on mathematics and statistics, employs quantitative analysis on specific knowledge carriers, which is scientific [18]. It requires a substantial amount of scientific data to provide a clear demonstration of the focus on the current status of research, hotspots, and frontiers in the research field [19, 20]. Even though an increasing number of scholars have begun to focus on palliative care within CRC research, there is a lack of bibliometric analysis on massive datasets. In order to identify the research hotspots in this field and understand how the research trend is developing, our study leverages bibliometric to systematically analyze literature related to palliative care in CRC research, aiming to provide theoretical insights and support further development in the field.

## Materials and methods

### Data collection

Literature retrieval was conducted on April 15, 2025, targeting studies published between January 1, 2015 and April 15, 2025. The data was obtained from the Web of Science Core Collection (WoSCC) (Science Citation Index Expanded). The search strategy followed this search formula: ((((TS=(“Bereavement Care” OR “Hospice Program*” OR “Hospice care” OR “Palliative care” OR “Supportive Care” OR “Palliative Treatment” OR “Palliative Therapy” OR “Palliative Surgery”)) AND TS=(“Colorectal Neoplasm*” OR “Colorectal Tumor*” OR “Colorectal Cancer*” OR “Colorectal Carcinoma*” OR “Colonic Neoplasm*” OR “Colon Neoplasm*” OR “Cancer of Colon” OR “Colon Cancer*” OR “Cancer of the Colon” OR “Colonic Cancer*” OR “Colon Adenocarcinoma*” OR “Rectal Neoplasm*” OR “Rectum Neoplasm*” OR “Rectal Tumor*” OR “Cancer of Rectum” OR “Rectum Cancer*” OR “Cancer of the Rectum” OR “Rectal Cancer*”)) AND DOP=(2015-01-01/2025-04-15)) AND DT=(Article OR Review: Articles and reviews are the mostwidely recognized and formally peer-reviewed types of scientific research outputs. In contrast,other document types (e.g., letters, conference abstracts) often lack rigorous peer review, haverelatively concise content, and exhibit insufficient citation frequencies. Including such typeswould undermine the accuracy and reliability of our bibliometric analysis; therefore, they wereexcluded from the search scope.)) AND LA=Rationale for excluding non-English articles: This decision is primarily based on twoconsiderations. On one hand, it ensures the consistency of language processing throughout ourstudy, avoiding potential biases or errors caused by multilingual differences. On the other hand,the analytical tool employed in this research has limited capabilities for processing non-Englishtexts. Moreover, as the dominant language in international scientific communication, Englishliterature has a more extensive citation base, which is conducive to ensuring therepresentativeness of our data.(English) (manual review). All retrieved articles were saved in plain text format and exported as full records.

### Data analysis

The methodological framework of this study was based on the approach described by Yan et al. [21]. Annual publication trends were visualized using Origin 2018. Data analysis was conducted with R software (version 4.4.3, RRID: SCR_001905) in conjunction with the bibliometrix package (version 4.0, http://www.bibliometrix.org) [22], as well as VOSviewer (version 1.6.18, RRID: SCR_023516) [23] and CiteSpace (version 6.1.4, RRID: SCR_025121) [24]. To ensure data accuracy and analytical consistency, we independently performed data extraction and analysis.

The bibliometrix package was applied to visualize and map the structure of scientific knowledge. VOSviewer was used to construct visual networks of country and institutional co-authorship, source co-citation, and keyword co-occurrence. A minimum of 5 publications was required for countries or institutions to be included in the co-authorship network. In the co-citation analysis, only sources with at least 40 citations were analyzed. For the keyword co-occurrence analysis, keywords with a minimum frequency of 10 were considered, excluding general terms such as “palliative care,” “colorectal cancer,” and their synonyms to improve analytical precision [25]. Journal impact factors (IFs) were obtained from the 2023 edition of the Journal Citation Reports (JCR).

## Results

### Overview of selected studies on palliative care in colorectal cancer

**Fig. 1:**
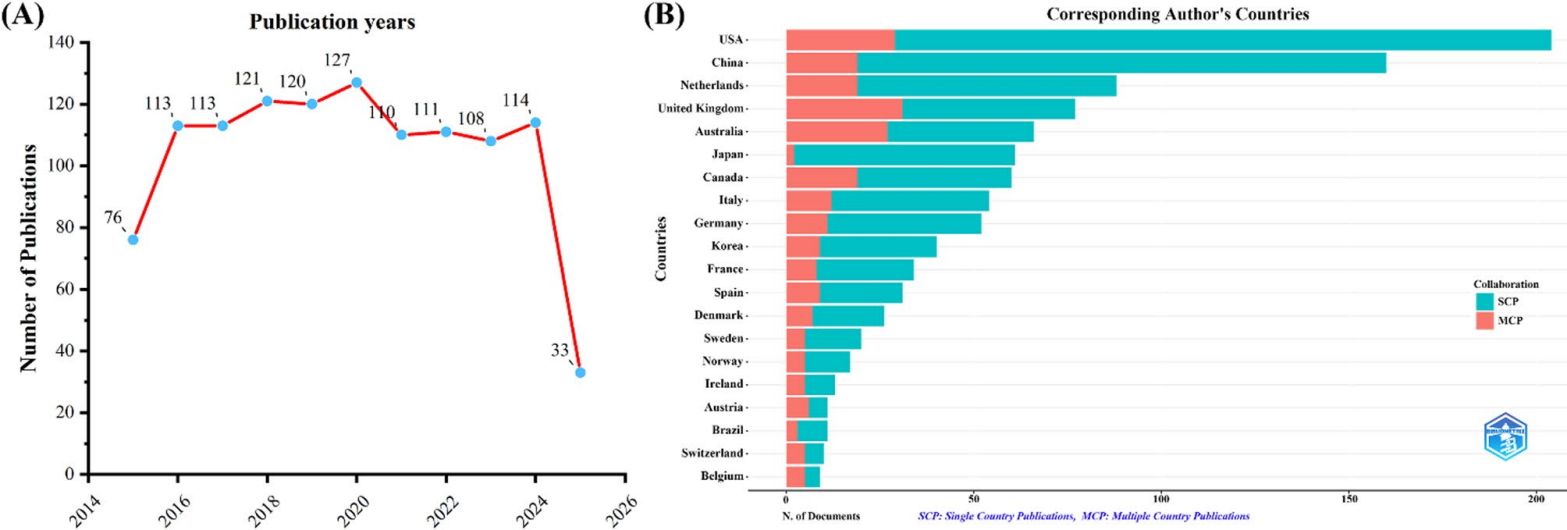
Trends in annual publication outputs related to palliative care in colorectal cancer (January 1, 2015 - April 15, 2025). (**A**) Depicts the annual publication trends. (**B**) Illustrates the distribution of countries and the collaborative efforts among corresponding authors

After removing duplicate records, a total of 1146 unique publications were retrieved from the WoSCC database. Between 2015 and 2016, the number of studies focusing on palliative care in CRC demonstrated an upward trend. From 2016 to 2024, the publication volume remained relatively stable, with a slight peak observed in 2020 (*n* = 127) (Fig. 1A), potentially reflecting the impact of the COVID-19 outbreak during that year [26]. In 2025, up to April 15, 33 relevant publications have been published.

**Fig. 2:**
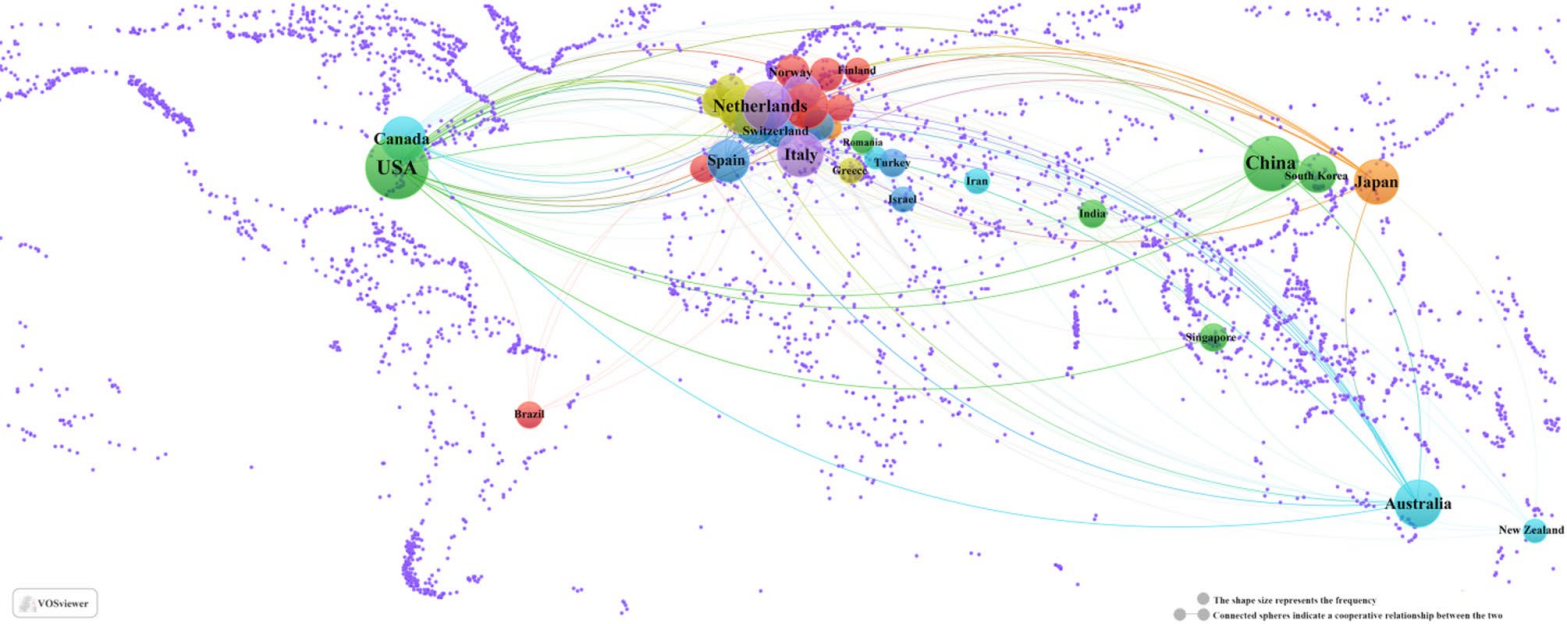
A map of countries involved in the field of palliative care in colorectal cancer (January 1, 2015 - April 15, 2025)

**Fig. 3:**
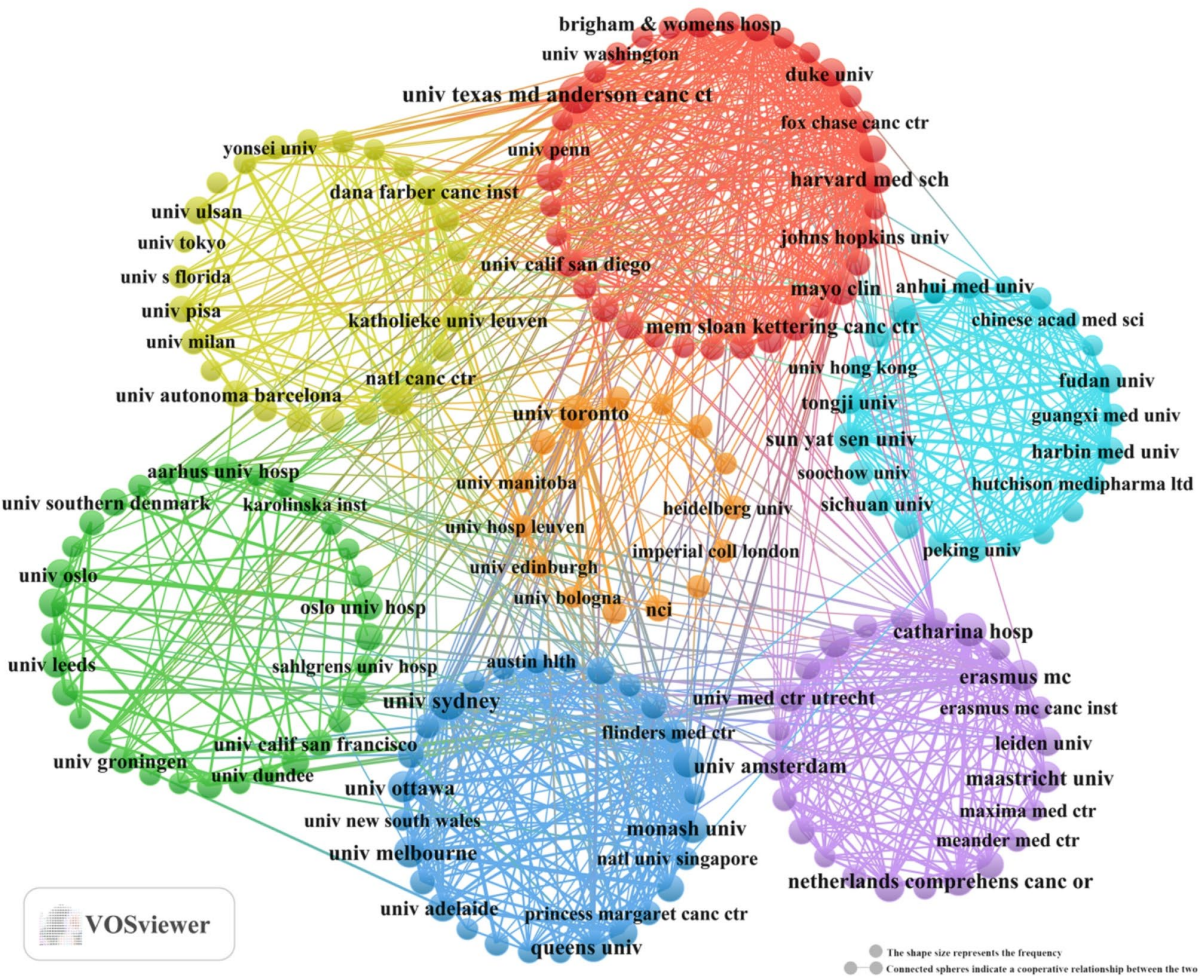
A map of institutions involved in the field of palliative care in colorectal cancer (January 1, 2015 - April 15, 2025)

Geographical analysis of corresponding authors revealed that the United States was the leading contributor (*n* = 204), followed by China (*n* = 160), the Netherlands (*n* = 88), the United Kingdom (*n* = 77), and Australia (*n* = 66). Notably, among the top five publishing countries, Australia exhibited the highest proportion of multi-country collaborations (MCPs), accounting for 40.9%, as illustrated in Fig. 1B and detailed in Table 1. As depicted in Fig. 2, the United States serves as a central hub for international collaboration in this field. Interestingly, no single institution demonstrated absolute dominance in terms of publication output (Fig. 3; Table 2).

**Table 1:** Most relevant countries by corresponding authors of palliative care in colorectal cancer (January 1, 2015 - April 15, 2025)

Country	Articles	Articles %	SCP	MCP	MCP %
USA	204	17.8	175	29	14.2
China	160	14	141	19	11.9
Netherlands	88	7.7	69	19	21.6
United Kingdom	77	6.7	46	31	40.3
Australia	66	5.8	39	27	40.9
Japan	61	5.3	59	2	3.3
Canada	60	5.2	41	19	31.7
Italy	54	4.7	42	12	22.2
Germany	52	4.5	41	11	21.2
Korea	40	3.5	31	9	22.5
France	34	3	26	8	23.5
Spain	31	2.7	22	9	29
Denmark	26	2.3	19	7	26.9
Sweden	20	1.7	15	5	25
Norway	17	1.5	12	5	29.4
Ireland	13	1.1	8	5	38.5
Austria	11	1	5	6	54.5
Brazil	11	1	8	3	27.3
Switzerland	10	0.9	5	5	50
Belgium	9	0.8	4	5	55.6

Note: MCP: Multiple country publication; SCP: Single country publication

**Table 2:** Top 10 most relevant affiliations of palliative care in colorectal cancer (January 1, 2015 - April 15, 2025)

Rank	Affliation	Articles (*n*)
1	univ sydney	32
2	univ texas md anderson canc ctr	29
3	mayo clin	23
4	univ toronto	22
5	catharina hosp	20
6	natl canc ctr hosp east	19
7	erasmus mc	17
8	harvard med sch	17
9	sun yat sen univ	17
10	univ amsterdam	17

### Journal analysis and visualization

To identify the journals that have made the most significant contributions in terms of publication volume and citation impact within the field of palliative care in CRC, the bibliometrix package in R was employed. Visualizations were generated using the ggplot2 package. Furthermore, journal co-citation analysis was conducted using VOSviewer.

**Fig. 4:**
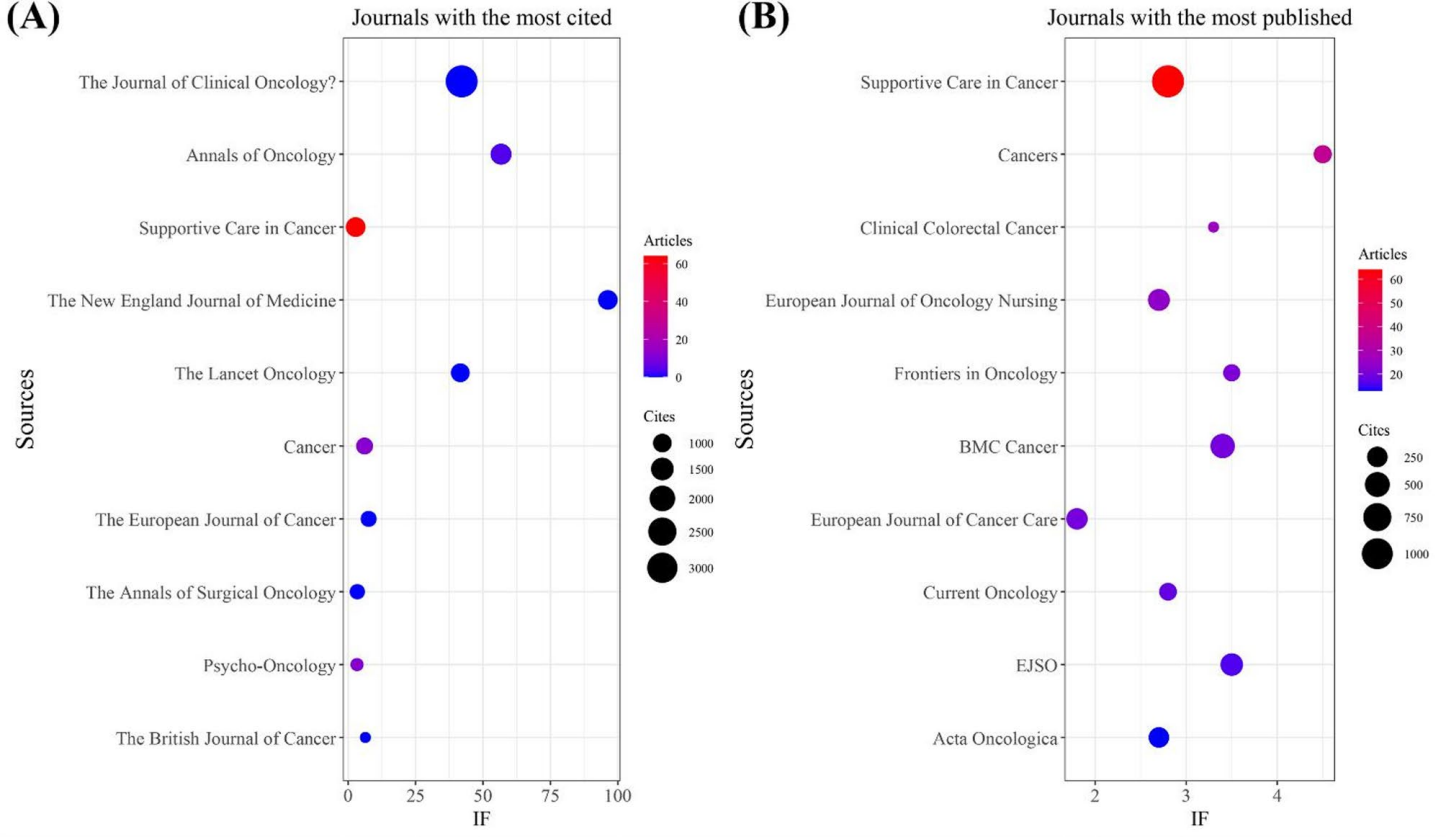
The journal with the highest volume of published articles and the journal with the most extensive citation count (January 1, 2015 - April 15, 2025). (**A**) The journal with the highest quantity of published documents. (**B**) The journals with the most substantial citation counts

**Fig. 5:**
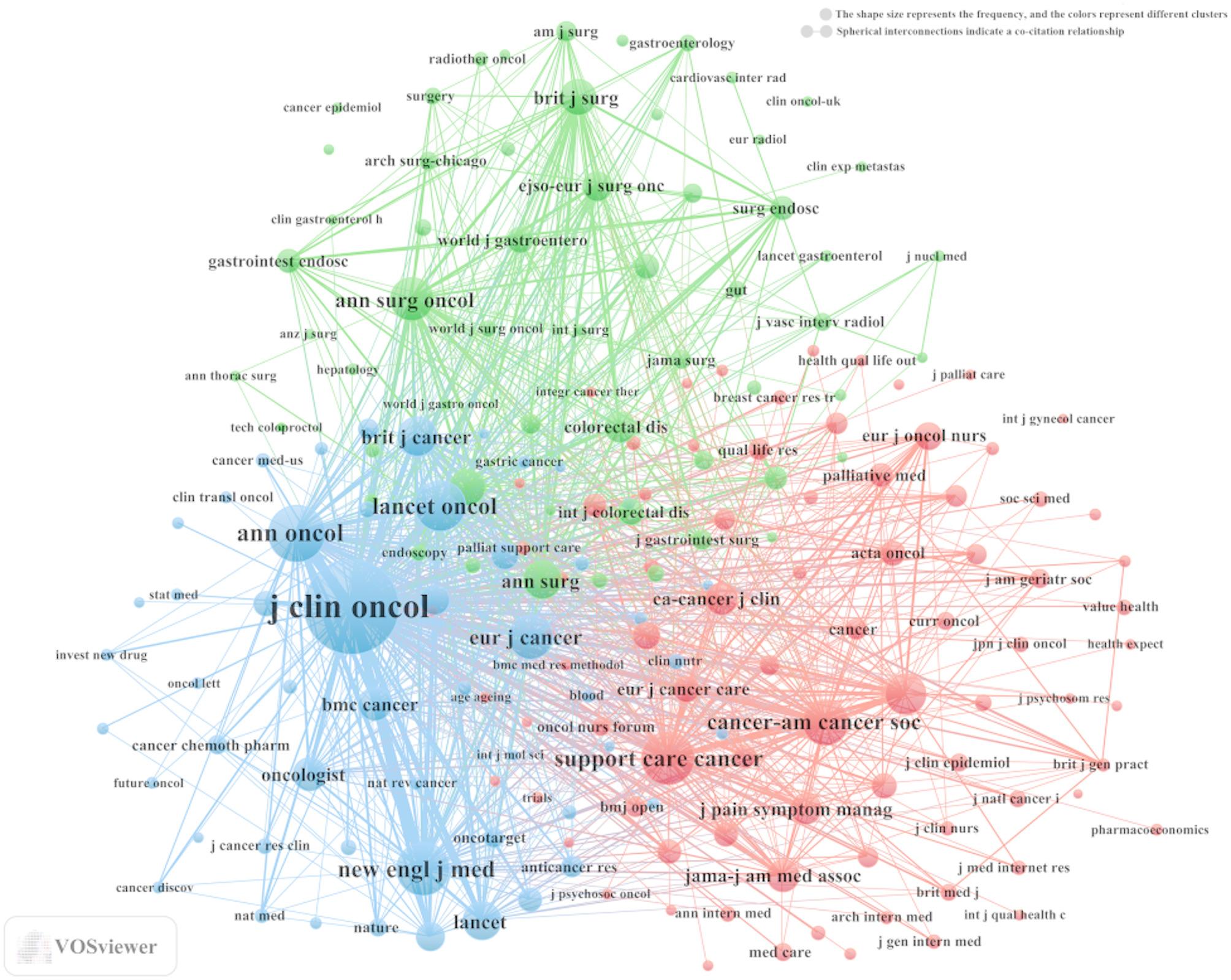
Co-cited journals related to palliative care in colorectal cancer (January 1, 2015 - April 15, 2025)

Our analysis identified a total of 1146 documents published across 363 academic journals (see Annex 1 for detailed information). As summarized in Table 3 and visualized in Fig. 4A, *Supportive Care in Cancer* (*n* = 64, IF = 2.8) ranked as the most prolific journal, followed by *Cancers* (*n* = 36, IF = 4.5), *Clinical Colorectal Cancer* (*n* = 26, IF = 3.3), *European Journal of Oncology Nursing* (*n* = 24, IF = 2.7), and *Frontiers in Oncology* (*n* = 21, IF = 3.5). Table 4; Fig. 4B present the most frequently cited journals, with *The Journal of Clinical Oncology* (*n* = 3433, IF = 42.1), *Annals of Oncology* (*n* = 1265, IF = 56.7), *Supportive Care in Cancer* (*n* = 1112, IF = 2.8), *The New England Journal of Medicine* (*n* = 1102, IF = 96.3), and *The Lancet Oncology* (*n* = 1018, IF = 41.6) leading the citation landscape. Furthermore, the co-citation network depicted in Fig. 5 identifies *The Journal of Clinical Oncology* (Total link strength:137056), *Supportive Care in Cancer* (Total link strength:37321), and *The Annals of Surgical Oncology* (Total link strength: 31977) as central hubs of scholarly collaboration. It is worth noting that although *The Journal of Clinical Oncology* and *The Annals of Surgical Oncology* form part of the citation core, the number of articles they have published in this field remains relatively limited. In contrast, *Supportive Care in Cancer* not only belongs to the citation core but also ranks as the most prolific journal in terms of publication volume. Therefore, researchers in this field may consider *Supportive Care in Cancer* a key source for relevant literature and future submissions.

**Table 3:** Top 10 journals with the most published (January 1, 2015- April 15, 2025)

Sources	Documents	IF(2023)	Cites
Supportive Care in Cancer	64	2.8	1112
Cancers	36	4.5	147
Clinical Colorectal Cancer	26	3.3	20
European Journal of Oncology Nursing	24	2.7	317
Frontiers in Oncology	21	3.5	115
BMC Cancer	20	3.4	467
European Journal of Cancer Care	20	1.8	288
Current Oncology	18	2.8	134
EJSO	16	3.5	353
Acta Oncologica	13	2.7	246

**Table 4:** Top 10 journals with the most cited (January 1, 2015 - April 15, 2025)

Sources	Cites	IF(2023)	Documents
The Journal of Clinical Oncology	3433	42.1	0
Annals of Oncology	1265	56.7	4
Supportive Care in Cancer	1112	2.8	64
The New England Journal of Medicine	1102	96.3	0
The Lancet Oncology	1018	41.6	0
Cancer	853	6.1	12
The European Journal of Cancer	778	7.6	0
The Annals of Surgical Oncology	738	3.4	0
Psycho-Oncology	654	3.3	12
The British Journal of Cancer	626	6.4	0

Note: citation counts represent references to articles from that journal within the dataset, not articles published by the journal on the specific topic

### Citation analysis

We utilized the bibliometrix package in R software to identify the top 20 most-cited references in the field of palliative care in CRC (Table 5). The top three cited papers were: “Practical assessment and management of vulnerabilities in older patients receiving chemotherapy: ASCO guideline for geriatric oncology”, “Consolidative radiotherapy for limited metastatic non–small-cell lung cancer: a phase 2 randomized clinical trial”, and “Regorafenib plus best supportive care versus placebo plus best supportive care in Asian patients with previously treated metastatic CRC (CONCUR): a randomised, double-blind, placebo-controlled, phase 3 trial”.

**Fig. 6:**
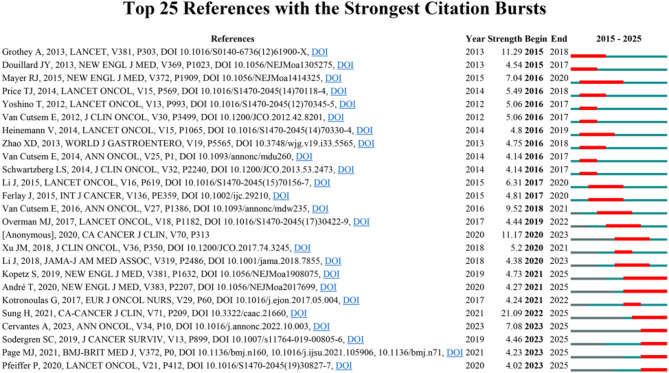
The top 25 most cited references on palliative care in colorectal cancer (January 1, 2015 - April 15, 2025)

To further investigate the cutting-edge developments and focal areas of palliative care in CRC research, we employed CiteSpace to identify the top 20 most prominent citation bursts associated with this topic (Fig. 6). The titles of these highly cited works, along with their corresponding DOIs, are detailed in Annex 2. Notably, the three citations with the strongest citation bursts were: (1) “Global cancer statistics 2020: GLOBOCAN estimates of incidence and mortality worldwide for 36 cancers in 185 countries (strength: 21.09)”; (2) “Regorafenib monotherapy for previously treated metastatic colorectal cancer (CORRECT): an international, multicentre, randomised, placebo-controlled, phase 3 trial (strength: 11.29)”; (3) “ESMO consensus guidelines for the management of patients with metastatic colorectal cancer (strength: 9.52)”. Furthermore, the titles of the three most cutting-edge citation bursts were: (1) “TAS-102 with or without bevacizumab in patients with chemorefractory metastatic colorectal cancer: an investigator-initiated, open-label, randomised, phase 2 trial”. (2) “PRISMA 2020 explanation and elaboration: updated guidance and exemplars for reporting systematic reviews”. (3) “Supportive care needs of patients following treatment for colorectal cancer: risk factors for unmet needs and the association between unmet needs and health-related QOL—results from the ColoREctal Wellbeing (CREW) study”.

Overall, through the most cited references and reference burst analysis, we have identified three key areas of focus within the field of palliative care in CRC: (1) Novel systemic palliative pharmacological therapies in CRC, (2) palliative interventional management of malignant bowel obstruction, (3) symptom monitoring and supportive care pathways, and exercise interventions and perioperative prehabilitation.

**Table 5:** The top 20 cited references related to palliative care in colorectal cancer (January 1, 2015 - April 15, 2025)

Paper	DOI	Total Citations	TC per Year
Mohile SG, 2018, J CLIN ONCOL	10.1200/JCO.2018.78.8687	961	120.13
Iyengar P, 2018, JAMA ONCOL	10.1001/jamaoncol.2017.3501	799	99.88
Li J, 2015, LANCET ONCOL	10.1016/S1470-2045(15)70156-7	634	57.64
Cormie P, 2017, EPIDEMIOL REV	10.1093/epirev/mxx007	416	46.22
Pietrantonio F, 2015, EUR J CANCER	10.1016/j.ejca.2015.01.054	402	36.55
Rubin G, 2015, LANCET ONCOL	10.1016/S1470-2045(15)00205-3	400	36.36
Wright AA, 2016, JAMA-J AM MED ASSOC	10.1001/jama.2015.18604	340	34.00
Carli, 2020, JAMA SURG	10.1001/jamasurg.2019.5474	312	52.00
Chen EX, 2020, JAMA ONCOL	10.1001/jamaoncol.2020.0910	274	45.67
Brule SY, 2015, EUR J CANCER	10.1016/j.ejca.2015.03.015	271	24.64
Shah MA, 2017, JAMA ONCOL	10.1001/jamaoncol.2016.5580	228	25.33
Van Hooft JE, 2020, ENDOSCOPY	10.1055/a-1140-3017	197	32.83
Pfeiffer P, 2020, LANCET ONCOL	10.1016/S1470-2045(19)30827-7	168	28.00
Aston WJ, 2017, BMC CANCER	10.1186/s12885-017-3677-7	143	15.89
Zimmer P, 2018, SUPPORT CARE CANCER	10.1007/s00520-017-3875-5	142	17.75
Jonker DJ, 2018, LANCET GASTROENTEROL	10.1016/S2468-1253(18)30009-8	139	17.38
Neufeld NJ, 2017, FUTURE ONCOL	10.2217/fon-2016-0423	139	15.44
Maguire R, 2021, BMJ-BRIT MED J	10.1136/bmj.n1647	136	27.20
Dittus KL, 2017, PREV MED	10.1016/j.ypmed.2017.07.015	129	14.33
Dasari A, 2023, LANCET	10.1016/S0140-6736(23)00772-9	128	42.67

### Keyword clusters and evolution of themes

Keyword clusters are essential for rapidly grasping the main research themes and directions in a particular area. In our study, VOSviewer was used to identify 4321 keywords. Table 6 displays the top 20 keywords, each occurring more than 58 times, highlighting the prominent research focuses. The most frequently appearing keyword was “quality of life” (*n* = 258), followed by “chemotherapy” (*n* = 224), “survival” (*n* = 202), “surgery” (*n* = 152), and “clinical-trials” (*n* = 148).

**Fig. 7:**
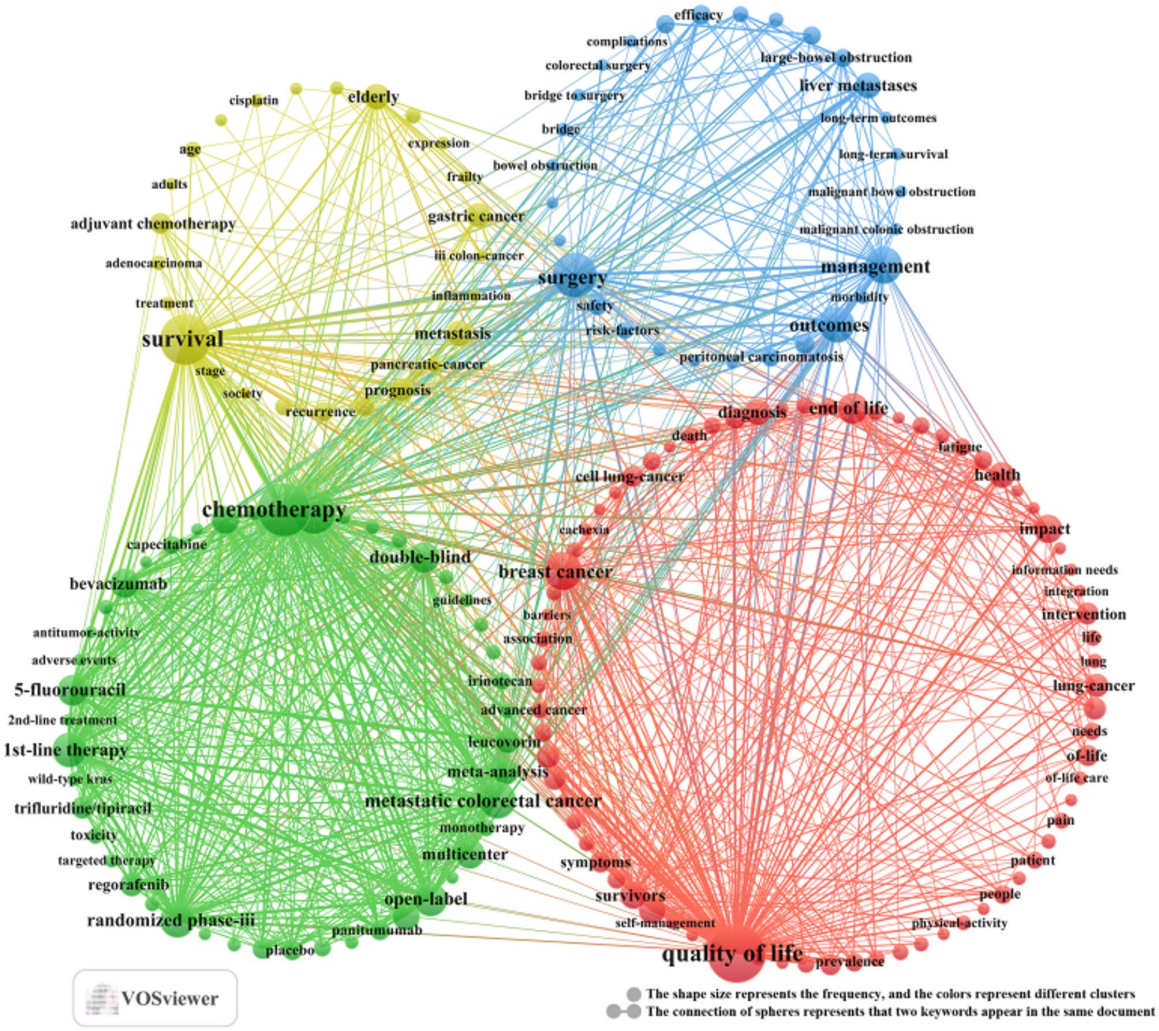
A co-occurrence map of keywords in the literature on palliative care in colorectal cancer (January 1, 2015 - April 15, 2025)

Through cluster analysis [21, 25], we observe four different colored clusters in Fig. 7. (1) Quality of life and psychosocial support needs in advanced patients with CRC (red dots), there are 74 keywords, including quality of life, symptoms, fatigue, unmet needs, information needs, and so on. (2) Systematic treatment and Individualized drug selection in palliative care for metastatic CRC (green dots), there are 46 keywords, including chemotherapy, bevacizumab, randomized phase-iii, 1st-line therapy, 5-fluorouracil, and so on. (3) Palliative surgical interventions: indications, strategies, and prognosis in advanced CRC (blue dots), there are 29 keywords, including palliative surgery, cytoreductive surgery, outcomes, efficacy, emergency-surgery, and so on. (4) Prognostic evaluation and adjuvant treatment decision-making in palliative care for elderly patients with CRC (yellow dot), there are 25 keywords, including elderly, survival, prognosis, adjuvant chemotherapy, radiation-therapy, and so on. All keywords contained in the four clusters can be found in Annex 3.

**Fig. 8:**
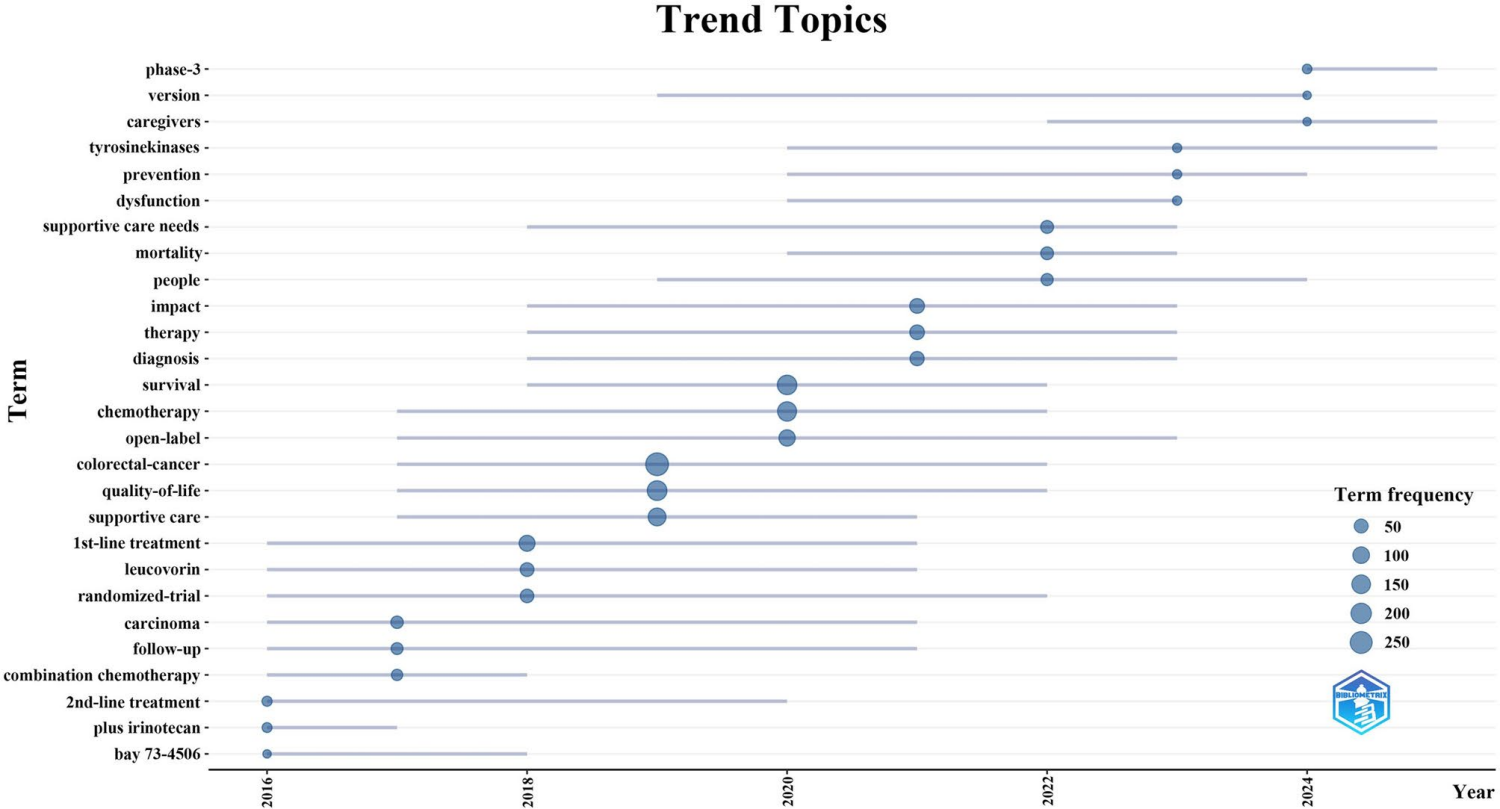
Trend topics on palliative care in colorectal cancer research (January 1, 2015 - April 15, 2025)

Furthermore, to anticipate emerging trends in this field, we employed the bibliometrix package in the R programming environment to generate a dynamic thematic evolution map (Fig. 8). From 2016 to April 15, 2025, the thematic evolution of palliative care research in CRC demonstrates a clear transition from treatment-centered approaches to more patient-centered, multidimensional care strategies. During the early phase (2016–2018), research primarily focused on optimizing chemotherapeutic regimens and treatment sequencing, with keywords such as “1st-line treatment,” “2nd-line treatment,” and specific agents like “leucovorin” and “bay 73-4506” indicating an emphasis on pharmacological strategies. Between 2017 and 2020, the scope broadened to encompass quality-of-life considerations and supportive care, as reflected by the emergence of terms such as “follow-up,” “supportive care,” and “quality-of-life,” signaling a shift toward evaluating the holistic impact of palliative interventions. In the subsequent period (2020–2023), research themes further diversified to address patient needs, mortality, diagnostic issues, and functional impairments, suggesting an increased awareness of the psychosocial and clinical complexity of palliative care. Most recently (2024–April 15, 2025), the focus has expanded to include precision medicine and caregiver support, with keywords like “tyrosine kinases,” “phase-3,” and “caregivers” highlighting the integration of novel therapeutics and recognition of caregiver roles in comprehensive palliative management. This temporal progression underscores an ongoing paradigm shift from disease-centered to integrative, patient- and family-oriented care models in the palliative treatment of CRC.

**Table 6:** The top 20 keywords related to palliative care in colorectal cancer (January 1, 2015 - April 15, 2025)

Rank	Keywords	Count
1	quality of life	258
2	chemotherapy	224
3	survival	202
4	surgery	152
5	clinical-trials	148
6	breast cancer	112
7	management	109
8	metastatic colorectal cancer	108
9	outcomes	96
10	randomized phase-iii	91
11	1st-line therapy	90
12	open-label	87
13	end of life	73
14	5-fluorouracil	71
15	double-blind	70
16	multicenter	66
17	bevacizumab	65
18	cetuximab	61
19	impact	59
20	oxaliplatin	58

### Comprehensive analysis of hotspots

In summary, our comprehensive analysis, incorporating citation burst detection, keyword frequency analysis, keyword clustering, and thematic evolution, has revealed emerging research frontiers at the intersection of palliative care in CRC. Our findings reveal that the research hotspots in this field are primarily focused on three key directions. (1) Precision and individualized systemic palliative treatment strategies:

This hotspot emphasizes the development of systemic pharmacological therapies tailored to the unique characteristics of patients with advanced or metastatic CRC, including age, comorbidities, molecular profiles, and drug tolerability. The integration of novel agents such as targeted therapies and immunotherapies into palliative regimens has attracted growing attention. Moreover, individualized drug selection and prognostic assessment—particularly in elderly patients—are essential for optimizing treatment efficacy and minimizing toxicity. (2) Palliative management of local complications and surgical interventions: This area highlights the importance of managing cancer-related complications such as malignant bowel obstruction, perforation, and bleeding through interventional or surgical means. The role of minimally invasive procedures, stent placement, and selective palliative surgeries has been increasingly studied in terms of their indications, safety, outcomes, and impact on patients’ QOL. Optimizing these interventions remains a critical aspect of comprehensive palliative care. (3) Multidimensional supportive care: symptom monitoring, psychosocial support, and rehabilitation: Comprehensive supportive care, centered on improving QOL, is a prominent and evolving research direction. This includes routine symptom assessment (e.g., pain, fatigue, appetite loss), psychological and psychosocial support services, structured care pathways, and integrative interventions such as exercise and perioperative prehabilitation programs. The growing adoption of digital symptom tracking tools [27] and multidisciplinary care models reflects a shift toward more personalized and holistic palliative care strategies.

## Discussion

### General information

In this study, we retrieved 1146 relevant papers on palliative care for CRC published from January 1, 2015, to April 15, 2025 through the WoSCC database, and analyzed data using R software, VOSviewer and CiteSpace. Between 2015 and 2016, the number of studies focusing on palliative care in CRC has shown an upward trend. From 2016 to 2024, the number of publications remained relatively stable, with a slight peak observed in 2020. It may be attributed to the following three reasons: (1) There are numerous treatment methods for early-stage of CRC, such as drug therapy, traditional Chinese medicine treatment, and surgery [28, 29]. Generally, stages I-II CRC could be resected with surgery, while palliative treatment is more frequently used for advanced CRC. (2) Advanced CRC presents more complications, including intestinal perforation, bleeding, severe pain and multiple metastases [28, 30], posing a significant challenge to patients’ physical condition. The low willingness and declining treatment adherence among most patients have increased resistance to further treatment, leading to prolonged study cycles. Additionally, some patients discontinued treatment, leading to an insufficient sample size, which made the study more challenging [31]. (3) Palliative care plays an essential role in improving the QOL for cancer patients, particularly those in advanced stages of the disease. Despite its proven benefits, attitudes toward palliative care vary widely among patients due to limited cognition cultural beliefs and personal values [32, 33].

In the field of palliative care research in CRC, the United States leads in publications, with China and the Netherlands following. This trend may be associated with healthcare resources and government-specific investments in the United States [34]. The NCCN guidelines indicate that palliative care should be integrated throughout the management of CRC, advancing the progress of related clinical trials [29].

At the same time, CRC is trending younger in the United States. Young patients have a high demand for QOL, which may also be driving palliative care research. A total of 1146 relevant articles were distributed across 363 academic journals, with prominent journals such as *Supportive Care in Cancer*,* Cancer*, and *Clinical Colorectal Cancer* contributing a large number of articles. Notably, *Supportive Care in Cancer* published a considerable number of papers and had a substantial number of citations, highlighting its role as the primary channel for dissemination of research achievements in this field.

### Emerging research frontiers at the intersection of palliative care in CRC

#### Precision and individualized systemic palliative treatment strategies

CRC is a highly prevalent tumor worldwide, currently ranking third in incidence. A study predicted an increase to 2.5 million cases of CRC worldwide by 2035 [35]. Currently, the common treatment for CRC involves a combination of surgery, radiotherapy, chemotherapy and targeted therapy. Relevant studies have shown that the choice of future treatment options for CRC primarily concentrates on personalized and targeted therapies [36]. Moreover, there is increasing attention to the integration of novel drugs such as targeted therapy and immunotherapy into palliative care [37]. Interventions include precision cancer medicine, targeted chemotherapy using nanocarriers, palliative care plus pressurized intraperitoneal aerosol chemotherapy (PIPAC), adjuvant lysosomal viral therapy and radioembolization techniques [38]. Based on individual genetics, comprehensive genetic analysis provides specific tumor-targeted therapy [39]. Moreover, as chemotherapy delivery systems, bionic magnetic nanoparticles can reduce the systemic side effects of conventional chemotherapy by targeting tumor cells and preserving healthy cells [40]. PIPAC is an emerging option for targeted therapy in patients with peritoneal metastases and is now being used internationally, showing encouraging outcomes.

Additionally, individualized drug selection and prognostic assessment—particularly in elderly patients—are essential for optimizing treatment efficacy and minimizing toxicity. CRC is one of the leading causes of cancer-related death among the elderly. Elderly patients with CRC often perform poorly in clinical trials, resulting in inadequate clinical treatment [41]. Advanced age should not be the sole criterion for excluding older patients with CRC from receiving effective treatment. A study indicated that elderly patients with CRC could benefit from adjuvant chemotherapy with 5-fluorouracil/ leucovorin or capecitabine to the same extent as younger patients, without a significant increase in toxicity [42]. The Comprehensive Geriatric Assessment (CGA) is a multidimensional evaluation that considers the elderly’s comorbidities and medication treatment, nutritional status, physical performance, and emotional function. Individualized CGA and geriatric interventions, including adjustments in medication, nutritional therapy, and exercise, have been shown to beneficial for patients with CRC receiving palliative care throughout the cancer treatment trajectory [43]. Due to the limited availability of medical resources, precision and individualized palliative care may be increasingly extend beyond the hospital in the future [44], encompassing community medical service centers and online medical services. It would be crucial to develop sufficient medical talents and transform traditional medical treatment model in future development.

#### Palliative management of local complications and surgical interventions

In recent years, the roles of minimally invasive procedures, stent placement, and selective palliative surgeries have been increasingly studied in terms of their indications, safety, outcomes, and impact on QOL of patient. Many patients with advanced CRC often develop acute intestinal obstructions. Traditional enterostomy is traumatic and has a poor prognosis, while postoperative artificial anal defecation also affects the patient’s QOL. Dohmoto et al. [45] who first reported the clinical application of metal stents for the treatment of malignant intestinal obstruction in 1991. Recently, there has been an increasing application in clinical treatments due to the combination of improved self-expandable metallic stent materials and endoscopic techniques. Studies have confirmed that metal stent placement, when compared to traditional fistula surgery, had a shorter operation time (with an average of approximately 40 min), a lower rate of postoperative complications, and reduced hospitalization costs [46]. These factors significantly alleviate the patient’s financial burden and yield positive social benefits [46]. The ESGE guidelines also recommend intestinal stenting as an optional treatment for acute intestinal obstruction, either temporarily or permanently for the palliative care of advanced cancerous obstructions [47]. Notably, this new minimally invasive procedure carries a risk of perforation [48]. It could involve a foreign body reaction between the stent material and the intestinal tissue [49]. Therefore, it may become a new development trend to find new degradable stent materials and optimizing the support strength of stents. This could not only reduce the incidence of postoperative complications, but also enhance the willingness of patients and their families to undergo treatment.

#### Multidimensional supportive care (symptom monitoring, psychosocial support, and rehabilitation)

For cancer patients, symptoms such as pain, loss of appetite, fatigue, and anxiety have become major problems that affect their QOL [51]. Regardless of whether the patient has undergone tumor-targeted therapy, palliative care is considered a recommended treatment option. Symptom-specific therapeutic care for those with incurable cancer deserves to be recognized. Existing studies have found that severe pain affects the QOL of patients with CRC receiving palliative care, and effective pain management and close nursing are necessary to alleviate pain [52]. A comprehensive review of the literature suggested, multimodal prehabilitation (exercise, nutritional, and psychological interventions) has been widely used in patients with CRC undergoing surgery and has improved clinical outcomes [53]. In psychotherapy, a randomized controlled trial utilizing web-based data demonstrated a reduction in depression among patients with CRC following psychological intervention [54]. As new techniques continue to be developed to help patients with cancer, music therapy is emerging as a prominent approach. The American Music Therapy Association suggested that individualized treatment goals would be achieved through clinical and evidence-based approaches. A study found that music therapy reduced pain, anxiety, and stress, and improved QOL among patients undergoing treatment for CRC [55]. It is evident that comprehensive supportive care, which focuses on improving QOL, is a prominent and evolving research direction. This shift is toward more personalized and multidimensional models of care.

The World Health Organization’s (WHO) public health approach to palliative care complements these three key areas, collectively contributing to the development of a multi-level palliative care system. The WHO approach emphasizes the importance of systematic integration, policy support, and efficient resource allocation by embedding palliative care within national health systems, fostering interdisciplinary collaboration, and encouraging community engagement [56]. For instance, the WHO’s community participation model synergizes with family-based support systems within the framework of comprehensive, multi-dimensional supportive care.

### Limitations

Based on the WOSCC database, this study reveals the current research status, hotspots, and development trends of palliative care for CRC through bibliometric and visualization analyses, which provides scientific value for the subsequent and continuous exploration of the field. However, our study also has limitations: Firstly, although the WOSCC database covers high-quality literature and is recognized as an ideal database for bibliometric analysis, nearly all the publications included in this study were in English with a lack of literature in other languages, which may cause the bias of language. Secondly, a single database includes limited literature, which could miss important research from different databases. Thirdly, the database is constantly updated, with changes in keyword indexing over time and annual variations in magazine influence, which may bias the study’s results. Furthermore, some high-quality literature may not be highlighted due to infrequent citation, and the literature included lacks an overall quality assessment, which inevitably leads to certain limitations in the results.

## Conclusion

This study outlines the current research perspective and potential areas of focus, offering a reference for trends and hotspots in palliative care research related to CRC. Personalized, multidisciplinary palliative care is likely to become a major focus of subsequent research in CRC. However, the social acceptance of palliative care needs to be improved to facilitate subsequent multicenter and large-sample studies.

## Supplementary Information

Below is the link to the electronic supplementary material.

Supplementary Material 1

Supplementary Material 2

Supplementary Material 3

## Data Availability

The raw dataset can be obtained from Web of Science using the provided search strategy. Processed data and supplementary materials are available in Annex 1, Annex 2 and Annex 3.
